# Lessons Learned From a Case of Gastric Cancer After Liver Transplantation for Hepatocellular Carcinoma: A Case Report and Literatures Review

**DOI:** 10.1097/MD.0000000000002666

**Published:** 2016-02-18

**Authors:** Kun Yang, Hong Zhu, Chong-Cheng Chen, Tian-Fu Wen, Wei-Han Zhang, Kai Liu, Xin-Zu Chen, Dong-Jiao Guo, Zong-Guang Zhou, Jian-Kun Hu

**Affiliations:** From the Department of Gastrointestinal Surgery (KY, HZ, W-HZ, KL, X-ZC, D-JG, Z-GZ, J-KH); Laboratory of Gastric Cancer (KY, W-HZ, KL, X-ZC, D-JG, J-KH); Department of Nephrology (C-CC); and Department of Liver Surgery and Liver Transplantation Center (T-FW), West China Hospital, Sichuan University, Chengdu, Sichuan Province, China.

## Abstract

Nowadays, de novo malignancies have become an important cause of death after transplantation. According to the accumulation of cases with liver transplantation, the incidence of de novo gastric cancer is anticipated to increase among liver transplant recipients in the near future, especially in some East Asian countries where both liver diseases requiring liver transplantation and gastric cancer are major burdens. Unfortunately, there is limited information regarding the relationship between de novo gastric cancer and liver transplantation. Herein, we report a case of stage IIIc gastric cancer after liver transplantation for hepatocellular carcinoma, who was successfully treated by radical distal gastrectomy with D2 lymphadenectomy but died 15 months later due to tumor progression. Furthermore, we extract some lessons to learn from the case and review the literatures.

The incidence of de novo gastric cancer following liver transplantations is increasing and higher than the general population. Doctors should be vigilant in early detection and control the risk factors causing de novo gastric cancer after liver transplantation. Curative gastrectomy with D2 lymphadenectomy is still the mainstay of treatment for such patients. Preoperative assessments, strict postoperative monitoring, and managements are mandatory. Limited chemotherapy could be given to the patients with high risk of recurrence. Close surveillance, early detection, and treatment of posttransplant cancers are extremely important and essential to improve the survival.

## INTRODUCTION

Liver transplantations have been well accepted as an effective treatment for end-stage liver diseases in China. A total of 20,877 patients had liver transplantations in Mainland China from 1980 to 2011.^1^ With the increasing number of liver transplantation and prolonged long-term survival, the risk of developing de novo cancers is also increased among liver transplant recipients.^2^ Nowadays, de novo malignancies have become an important cause of death after transplantation.^2^ It has been reported that de novo cancers were the second most frequent etiology of death in transplant recipients.^3^ Various cancers such as skin tumors, Kaposi sarcoma, lymphoma, lung cancer, thyroid cancer, and brain tumor could be developed, in which skin tumors and lymphoproliferative malignancies are most frequent.^4,5^ Gastric cancer is not very common after liver transplantation. However, according to the accumulation of cases with liver transplantation, the incidence of de novo gastric cancer is anticipated to increase among liver transplant recipients in the near future, especially in China where both liver diseases requiring liver transplantation and gastric cancer are still major burdens. Unfortunately, there is only limited knowledge regarding the relationship between de novo gastric cancer and liver transplantation. Herein, we report a case of gastric cancer after liver transplantation for hepatocellular carcinoma, who was successfully treated by radical distal gastrectomy with D2 lymphadenectomy, extract some lessons we can learn from the case, and review the literatures. The patient written informed consent was waived due to the retrospective nature, and the patient information was anonymized.

## CASE PRESENTATION

**FIGURE 1:**
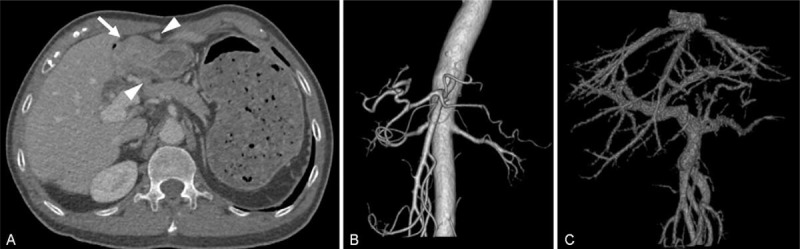
Images of 3-dimensional computed tomography (A, thickened antral wall (*arrow*) with multiple enlarged perigastric lymph nodes (*arrow head*) and no recurrence of hepatocellular carcinoma and distal metastasis were identified. B, There was no gross arterial anomaly around the stomach and hepatoduodenal ligament. C, The portal vein and its right branch were slightly dilated).

A 63-year-old man presented to our out-patient department with epigastric pain for 3 months. He had a history of cadaveric liver transplantation due to chronic hepatitis B related hepatocellular carcinoma 8 years ago, and cyclosporine and mycophenolate mofetil were administered for immunosuppression after surgery. Subsequently, however, he defaulted follow-up to the out-patient clinic. He had no history of gastric disease and smoking-alcohol abuse. Physical examination did not reveal any abdominal mass or enlargement of Virchow lymph nodes. A rectal digital examination was also unremarkable. His routine biochemistry, blood count, and tumor markers including alpha fetal protein (AFP), carcinoembryonic antigen (CEA), and carbohydrate antigen 19-9 (CA19-9) were within normal ranges. Breath test of Helicobacter Pylori (Hp) showed positive results. Gastroscopy revealed a deep ulcer with an irregular raised margin located at the greater curvature of gastric antrum, and histological examination of the biopsied specimen showed poorly differentiated adenocarcinoma. Three-dimensional computed tomography (CT) and magnetic resonance imaging (MRI) demonstrated thickened antral wall with multiple enlarged perigastric lymph nodes; no recurrence of hepatocellular carcinoma and distant metastasis were identified. There was no gross arterial anomaly around the stomach and hepatoduodenal ligament, but the portal vein and its right branch were slightly dilated (Figure 1). Curative distal gastrectomy with D2 lymph node dissection and Billroth-II reconstruction were performed. Midline incision was used for laparotomy, revealing a huge mass (10 × 8 × 6 cm in diameter) at the greater curvature of gastric antrum with obvious No. 3 and No. 6 lymph nodes metastasis. No peritoneal seeding and liver metastasis were noted. The margins were proved negative by frozen section examination. Finally, pathological examination confirmed the diagnosis of stage IIIc gastric adenocarcinoma (pT4aN3bM0). A total of 42 lymph nodes were harvested with 18 showing nodal metastasis. Cyclosporine and mycophenolate mofetil were resumed 1 day after the operation through nasogastric tube route, and the concentrations of immunosuppressive agents and liver functions were monitored perioperatively. The patient's postoperative course was uneventful, and he was discharged on postoperative day 10 without any complications. Similar dosages of the immunosuppressive agents were used after the operation. Postoperative adjuvant chemotherapy was rejected by the patient and his families. The patient subsequently developed peritoneal and para-aortic lymph node recurrence (12 months after operation) and died 3 months later due to tumor progression.

## DISCUSSION

De novo malignancy after organ transplantation has become a serious problem, affecting the long-term survival of the recipients.^2^ Moreover, the incidence of de novo gastric cancers is expected to increase in patients who underwent liver transplantation. However, little is known about the occurrence of gastric cancer after liver transplantation. Under this context, we try to summarize some lessons learnt from our case.

### Lesson 1: Doctors Should Be Vigilant in Early Detection and Control the Risk Factors Causing de Novo Gastric Cancer After Liver Transplantation

The exact mechanism of de novo gastric cancer development after liver transplantation is not clear. However, several factors may account for the carcinogenesis. First, pretransplant disease status may contribute to the development of de novo gastric cancer after liver transplantation. It has been reported that a history of autoimmune liver disease may promote carcinogenesis of de novo cancer in liver transplantation patients.^3^ Second, the risk of de novo cancer in post-transplant recipients was higher in men than women.^5^ Age >40 years was associated with significantly increased risk.^6^ Third, infectious factors may play a role in the development of de novo gastric cancer after liver transplantation, as some cancers are associated with carcinogenic Epstein-Barr virus (EBV),^7,8^ and more and more studies are showing an association between EBV infection and gastric cancers.^9–11^ In addition, Hp infection causing chronic gastritis or peptic ulcers may also be a pathogenic factor.^12^ It has been reported that secreted peptidyl prolyl cis, trans-isomerase of Hp was an important factor to drive gastric Th17 response, which could promote proinflammatory low cytotoxic tumor-infiltrating lymphocytes response, matrix degradation, and proangiogenic pathways.^13^ Fourth, unhealthy life styles might predispose to the oncogenesis of de novo gastric cancer. Previous studies have reported that smoking, alcohol consumption, and poor eating habits were considered to increase the risk of developing de novo malignancy,^14–17^ due to the impairment of gastric mucosa.^18,19^ And alcohol is hypothesized to decrease natural killer cell activity and promote tumor metastasis.^17,20^ Finally, but the most importantly, immunosuppression treatment is strongly associated with the development of de novo gastric cancer when compared with the immunocompetent population.^16,21^ Long-term use of immunosuppressive drugs, such as thiopurines and Tacrolimus that are often used after transplantation, could increase the risk of cancer directly with unknown mechanisms.^22,23^ The immunosurveillance role of immune system in liver transplantation recipients was long-term suppressed by the probable lifelong immunosuppressive treatment, which attributes to tumorigenesis of de novo gastric cancer.^24,25^ Furthermore, immunosuppressive treatment also aggravates the susceptibility and infection of Hp and virus.

Although there are some uncontrollable factors, doctors should be vigilant and intervene early for the clinically correctable factors. The patients could be advised to eliminate their unhealthy life styles. EBV and Hp eradication treatments might be necessary to reduce the risk of a de novo gastric cancer in transplant recipients. In the present case, the results of Hp examination were positive. Hp eradication therapy might prevent the development of metachronous gastric cancer.^26^ Meanwhile, minimization of immunosuppressive therapy without compromising the protection to the transplanted liver or using new immunosuppressive agents with lower adverse effects might help in reducing the risk of a de novo neoplasm.

### Lesson 2: Curative Gastrectomy With D2 Lymphadenectomy Is Still the Mainstay of Treatment for Patients With de Novo Gastric Cancer After Liver Transplantation

Surgery is the mainstay of treatment for medically fit patients with de novo gastric cancer after liver transplantation although no standard treatment regimen has been recommended in the literature. Open surgery may be an optimal choice with respect to the adhesions and oncological safety. A midline incision for open laparotomy is common as it can expose the operative field well and facilitate avoidance of tissue adhesions.^16^ Having said that, some have reported laparoscopic gastrectomy with lymph node dissection was feasible and safe for gastric cancer in patients with liver transplantation.^27^ Laparoscopic surgery could result in minimization of tissue injury and fewer inflammatory and immunologic reactions compared with open surgery,^28,29^ which meant laparoscopic gastrectomy might be more suitable for liver transplantation recipients with gastric cancer in this respect. However, this procedure was technically demanding and challenging. For early gastric cancer without evidence of lymph node metastasis, endoscopic submucosal dissection may be another option.^30^

Although the lymphatic flow was changed after the liver transplantation, D2 lymphadenectomy that was proved to be benefit to prolong the long-term survival of gastric cancer patients should also be recommended for de novo gastric cancer. Lymph nodes (especially Nos. 5, 8a, and 12a), however, should be dissected very carefully with much attentions paid to the adjacent adhesions, reconstructed vessels, and bile ducts to avoid the iatrogenic injuries.^16,27^

### Lesson 3: Preoperative Assessments, Strict Postoperative Monitoring, and Managements Are Mandatory

The patient's medical history including the reason of transplantation, the surgical procedure of liver transplantation, and its impact on gastrectomy must be investigated prior to surgery. Liver function, activity of hepatitis virus, concentrations of immunosuppressive agents, recurrence of primary liver tumor, and distant metastasis of gastric cancer, etc., should be evaluated in detail. Importantly, the anatomical structures, especially in the hepatoduodenal ligament, might be disorganized by the conversions of blood vessels at the time of transplantation. Therefore, careful preoperative assessment on vascular anatomy is important for patients undergoing gastrectomy to reduce the serious problems.^31^ Three-dimensional CT or angiography might be beneficial to demonstrate the structure of blood vessels preoperatively. Multidisciplinary treatment decision-making by members of gastrointestinal surgery, hepatic-biliary-pancreatic surgery, medical oncology, gastroenterology, radiology, and pathology is effective and should be encouraged before and after the surgery.

Immunosuppressants and enteral nutrients could be administered early via a nasogastric tube.^16^ There is no strong evidence about how the immunosuppressive agents should be administered perioperatively. Similar to other reports, our case continued to use immunosuppressive agents until the operation day and resumed through tube feeding 1 day after the operation. Some researchers suggested that fluctuation of the concentration of immunosuppressive agents should be minimized during the operative period.^27^ And balance of immunological activity should be kept to prevent acute rejection without suppressing the antitumor activity excessively. Postoperative monitoring of liver function and concentration of immunosuppressants is necessary.

### Lesson 4: Limited Chemotherapy Could Be Given to the Patients With High Risk of Recurrence

With respect to the adjuvant chemotherapy, the results remain controversial. The role of adjuvant chemotherapy in improving the prognosis of gastric cancer patients after D2 gastrectomy has been well established.^32,33^ In another hand, chemotherapy may harm the liver and worsen the immunocompromised status. However, Park et al reported almost all the patients with de novo malignancies after liver transplantation in Korea could receive aggressive cancer treatment including surgery, chemotherapy, and radiotherapy.^34^ Postoperative adjuvant chemotherapy was rejected in this far advanced stage case, which may account for the reason of his fast recurrence. Therefore, limited systemic treatment could be given to the patients with advanced stage cancers and high risk of recurrence.

### Lesson 5: Close Surveillance, Early Detection, and Treatment of Post-Transplant Cancers Are Extremely Important and Essential to Improve the Survival

Since the long term survival after transplantation has been improved significantly by development of the new immunosuppressive agents, the risk of developing de novo malignancy is increased.^2^ The overall incidence of malignancy in the transplant recipients is about 20%.^2^ For liver transplantation recipients, there is a 2 to 4 fold increase in risk of developing cancer compared to the general population.^5,6,35^ For gastric cancer, Buell et al reported that among 7000 post-transplant de novo malignancies, 33 gastric cancers were identified, in which 3 cases were observed after liver transplantation.^36^ The incidence of de novo gastric cancer after liver transplantation was reported to range from 0.14% to 1% (Table 1).^4,17,34,35,37–41^ Patients received liver transplantation for hepatocellular carcinoma might have a higher risk due to genetic predisposition to malignancy.^42,43^

**TABLE 1:**
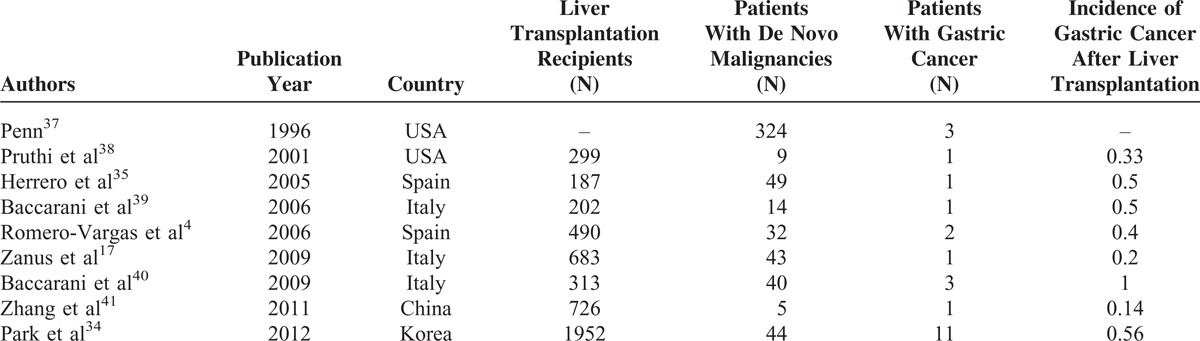
De Novo Malignancies After Liver Transplantation

However, the prognosis of patients with de novo malignancies in liver transplant recipients is usually worse than that in the general population. Continuation of immunosuppressive agents could be associated with poor prognosis for malignant tumor patients.^21^ Some systemic treatment was restricted as mentioned above, and transplant recipients generally have advanced stage cancers at the time of diagnosis.^44^ The reported 5-year survival rates of de novo gastric cancer after all kinds of transplantation were 67% in stage I/II and 8% in stage III,^36^ which were lower than those of general population.^45^ However, Buell et al^46^ found that the survivals of de novo gastric cancer in transplantation recipients were equivalent to that of general population since it was frequently identified at an earlier stage in comparison with the general population. Therefore, early detection and treatment of post-transplant cancers are extremely important. Considering the higher risk of developing malignancies after transplantations, close surveillance in such patients is essential to detect de novo tumors at early stage to improve the survival.^3,16,21,31^ Endoscopic examination might be prescribed and beneficial to the transplantation recipients with gastric symptoms.^36^ However, the present case did not obey the recommendation of doctor to visit the out-patient clinic regularly after liver transplantation, which eventually led to the gastric cancer being found in late stage with 10 cm in size. Further research regarding the optimal surveillance procedure is needed.

## CONCLUSIONS

In conclusion, the incidence of de novo gastric cancer after liver transplantations is increasing and higher than the general population. Doctors should be vigilant in early detection and control the risk factors causing de novo gastric cancer after liver transplantation. Curative gastrectomy with D2 lymphadenectomy is still the mainstay of treatment for such patients. Preoperative assessments, strict postoperative monitoring, and managements are mandatory. Limited chemotherapy could be given to the patients with high risk of recurrence. Close surveillance, early detection, and treatment of post-transplant cancers are extremely important and essential to improve the survival.
